# Qing-Re-Xiao-Zheng Formula Modulates Gut Microbiota and Inhibits Inflammation in Mice With Diabetic Kidney Disease

**DOI:** 10.3389/fmed.2021.719950

**Published:** 2021-09-16

**Authors:** Yabin Gao, Ruibing Yang, Lan Guo, Yaoxian Wang, Wei Jing Liu, Sinan Ai, Ting Hui Woon, Zheng Wang, Yuanyuan Zhai, Zhen Wang, Liang Peng

**Affiliations:** ^1^Department of Nephrology, Dongzhimen Hospital, Beijing University of Chinese Medicine, Beijing, China; ^2^The First Affiliated Hospital of Henan University of Traditional Chinese Medicine, Zhengzhou, China; ^3^Jitang College of North China University of Science and Technology, Hebei, China; ^4^Singapore General Hospital, Singapore, Singapore; ^5^College of Life Sciences, Hebei University, Hebei, China; ^6^Beijing Key Lab for Immune-Mediated Inflammatory Diseases, Department of Pharmacology, Institute of Clinical Medical Sciences, China-Japan Friendship Hospital, Beijing, China

**Keywords:** Qing-Re-Xiao-Zheng formula, gut microbiota, inflammation, diabetic kidney disease, TLR4/NF-κB pathway

## Abstract

Evidence indicates that the metabolic inflammation induced by gut microbiota dysbiosis contributes to diabetic kidney disease. Prebiotic supplementations to prevent gut microbiota dysbiosis, inhibit inflammatory responses, and protect the renal function in DKD. Qing-Re-Xiao-Zheng formula (QRXZF) is a Traditional Chinese Medicine (TCM) formula that has been used for DKD treatment in China. Recently, there are growing studies show that regulation of gut microbiota is a potential therapeutic strategy for DKD as it is able to reduce metabolic inflammation associated with DKD. However, it is unknown whether QRXZF is effective for DKD by regulating of gut microbiota. In this study, we investigated the reno-protective effect of QRXZF by exploring its potential mechanism between gut microbiota and downstream inflammatory pathways mediated by gut-derived lipopolysaccharide (LPS) in the kidney. High-fat diet (HFD) and streptozotocin injection-induced DKD mice model was established to assess the QRXZF effect *in vivo*. Mice treated with QRXZF for 8 weeks had significantly lower levels of urinary albumin, serum cholesterol and triglycerides. The renal injuries observed through histological analysis were attenuated as well. Also, mice in the QRXZF group had higher levels of Zonula occludens protein-1 (ZO-1) expression, lower levels of serum fluorescein-isothiocyanate (FITC)-dextran and less-damaged colonic mucosa as compared to the DKD group, implying the benefit role for the gut barrier integrity. QRXZF treatment also reversed gut dysbiosis and reduced levels of gut-derived LPS. Notably, the expression of toll-like receptor 4 (TLR4) and nuclear factor-κB (NF-κB), which are important inflammation pathways in DKD, were suppressed in the QRXZF groups. In conclusion, our results indicated that the reno-protective effects of QRXZF was probably associated with modulating gut microbiota and inhibiting inflammatory responses in the kidney.

## Introduction

According to the International Diabetes Federation (IDF) Diabetes Atlas 2019, nearly 10% of adults suffer from diabetes mellitus. This translates to 463 million people worldwide, and the number is expected to increase to 700.2 million (1). Diabetic kidney disease (DKD) is a common microvascular complication of diabetes, and remains as the main cause of end-stage renal disease (ESRD). However, there is no cure available and it has caused a large financial burden (2, 3).

Evidence suggests that gut microbiota has been implicated in the pathogenesis of several risk factors of DKD involving obesity, insulin resistance and diabetes (4–7). The gut microbiota helps to supply nutrients and vitamins, fights off invasive pathogens and protects intestinal barrier function (8–10). Accumulating studies have proposed gut-kidney axis plays great role in DKD by several gut-derived factors (11). Studies show that regulation of gut microbiota is a potential therapeutic strategy for DKD as it is able to reduce metabolic inflammation associated with DKD (12, 13). In particular, gut-derived endotoxins such as LPS, an inflammatory marker involved in the pathogenesis of DKD (14), Gut dysbiosis suppresses the expression of tight junction proteins, leading to increased intestinal permeability and the translocation of Gram-negative bacteria-derived LPS into the blood (15), that might be involved in metabolic inflammation and DKD progression (16, 17).

Prebiotic supplementations are non-digestible food ingredients, which play renal protective effect mainly by enhancing the growth of specific beneficial bacteria in the gut. Prebiotics not only alter the intestinal microbiota but also improve intestinal tight junction integrity and decrease blood endotoxemia caused by LPS. Traditional Chinese Medicine (TCM) is an alternative treatment for patients with DKD in China (18, 19). Qing-Re-Xiao-Zheng formula (QRXZF) which was formulated based on the “ZhengJia” theory in TCM has been commonly used for DKD treatment (20). It comprises of *Astragali radix IV* (Huang Qi), *Radix angelicae sinensis* (Dang Gui), *Concha Ostreae* (Mu Li), *Rheum officinale Baill* (Da Huang), and four other herbs. Since gut microbiota and inflammatory responses might lead to the progression of DKD (13, 14), our study aims to investigate the anti-inflammatory and reno-protective effects of QRXZF in DKD mice by observing alterations in gut microbiota and levels of gut-derived LPS and identifying the relationship between gut microbiota and DKD.

## Materials and Methods

### Herbal Formation and Component

QRXZF consists of *Astragali radix IV* (Huang Qi), *Radix angelicae sinensis* (Dang Gui), *Concha Ostreae* (Mu Li), *Rheum officinale Baill* (Da Huang), and four other herbs. Herbs were weighed and boiled at 10°C for 1 h and the final concentration was extracted into 2 g/ml. Herbs were purchased from Beijing Tong Ren Tang, which has high quality control standards validated according to the Chinese Pharmacopeia (China Pharmacopoeia Committee, 2015).

### Animals and Ethics Statement

Seven-week-old male C57BL/6J mice were purchased from Jiangsu-Jicui Yaokang Lab Animal Ltd. Mice in the control group were fed with common feed while mice in the high fat diet (HFD) group were fed with high fat food (60 kcal% fat, D12492, Research Diets, New Brunswick, NJ, United States). Mice were kept 3 per cage in specific pathogen-free (SPF) conditions, under controlled environmental conditions (a 12-12 h light-dark cycle, 22 ± 2°C room temperature, and 60-65% relative humidity while free access to water as well as food). All experimental procedures were approved by the Ethics Committee of Beijing University of TCM and performed following the “Guide for the Care and Use of Laboratory Animals” published by the National Institutes of Health.

### Experimental Design

After fasting for 16 h, mice in the HFD group fed for 7 weeks were treated daily with streptozotocin (STZ) (40 mg/kg/d, i.p; Sigma, USA) freshly dissolved in citrate buffer (0.1 mol/L, pH 4.3) for 5 days consecutively, while the control group were treated with citrate buffer.

Seven days after the last injection, blood glucose levels were tested by obtaining blood from the tail vein after an overnight fast. Mice with glucose levels over 16.7 mmol/L were randomly assigned to either the DKD (*n* = 6) or the QRXZF (*n* = 6) group. Mice in the DKD group were gavaged with saline water 0.25 ml/d, while mice in the QRXZF group were treated with QRXZF at a dose of 15.6 g/kg/d. Treatment was done via intragastric gavage daily for 8 weeks.

Fresh fecal samples were frozen in liquid nitrogen and stored at −80°C before further processing. Urine samples were collected with metabolic cages and stored at −20°C. 4 h before sacrifice, fluorescein-isothiocyanate (FITC)-dextran (44 mg/100 g, 4 kDa; Sigma), a high molecular weight glucose polymer, which cannot be digested, were fed to the mice to assess changes in intestinal permeability. Blood samples were collected without anticoagulants and centrifuged at 3,000 rpm for 15 min. Organs including kidney and colon issues were stored at −80°C before further analysis.

### Serum and Urine Biochemical Assays

Blood glucose levels were tested by One Touch Ultra 2 glucometer (Johnson, USA). Serum cholesterol, triglyceride, creatinine, and urine creatinine were measured using ELISA kits purchased from Nanjing Jiancheng Bioengineering Institute (Jiangsu, China). Urine albumin and serum LPS were detected using commercial assay kits (Bethyl Laboratories, USA) and (LONZA, USA), respectively. Serum was diluted in phosphate-buffered saline (PBS) (1:1) and analyzed for FITC-dextran concentration by a fluorescence spectrophotometer (485 nm excitation, 535 nm emission).

### Histological Examination

Kidney and colon tissues were fixed in 10% formalin, embedded in paraffin while cut into 2 μm-thick sections for staining. Kidney tissues were investigated after hematoxylin-eosin (HE) staining, Masson trichome staining as well as periodic acid-Schiff (PAS) staining. Colon tissues were investigated after HE staining. Histological analysis was conducted on 50 full-sized glomeruli obtained from each specimen after PAS-staining. The level of glomerulosclerosis was scored as follows: 0, no sclerosis; 1, sclerosis observed in <10% of glomeruli; 2, sclerosis observed in 10-25% of glomeruli; 3, sclerosis observed in 25–50% of glomeruli; 4, sclerosis observed in >50% of glomeruli.

### Western Blotting Analysis

Kidney and colon tissues from each mouse were homogenized in radioimmunoprecipitation assay (RIPA) buffer with protease inhibitors. The amount of protein in the samples were quantified using the Bradford assay and equal quantities of protein were separated by sodium dodecyl sulfate–polyacrylamide gel electrophoresis (SDS-PAGE). After protein transfer, membranes were blocked in 5% BSA for 1 h and incubated at 4°C overnight with specific primary antibodies, and then incubated with horseradish peroxidase (HRP) linked secondary antibody. Antibodies specific to Zonula occludens protein-1 (ZO-1) (ab96587), Toll-like receptor 4 (TLR4) (ab13867), nuclear factor-κB (NF-κB) p65 (ab16502) and phospho-NF-κBp65 (Ser536, ab86299) were purchased from Abcam.

### Microbiota Analysis

Total genome DNA was collected from fecal samples using the PowerSoil DNA Isolation Kit (MoBio Laboratories, Carlsbad, CA). Assessment of DNA quality was conducted with 1% agarose gel electrophoresis. 16S rRNA gene sequencing was performed on gut microbiota composition in the mice. The V3-V4 hypervariable regions of the 16S rRNA gene were amplified by universal primers 338F (5′-ACTCCTACGGGAGGCAGCA-3′) as well as 806R (5′-GGACTACHVGGG TWTCTAAT-3′) incorporating sample barcode sequences. After the quality assessment, the library was sequenced on the MiSeq platform (Illumina) to generate 300-bp paired-end reads. In order to obtain effective reads, the Trimmomatic software was used to filter the poor-quality reads. Chimera sequences were removed by using the UCHIME algorithm. The sequencing data were submitted to the National Center of Biotechnology Information (NCBI) Sequence Read Archive Database with the accession no.PRJNA729207.

Operational taxonomic units (OTUs) were identified as 1 cluster with the similarity cutoff of 97%. We used the Mothur software to plot the rarefaction curve. Chao1 index and observed_species indices were performed to quantify and compare the alpha diversity. The principal component analysis (PCA) analysis and the Non-metric multidimensional scaling (NMDS) were performed using QIIME (http:qiime.org/) to compare beta diversity. Bacterial taxa of the groups were analyzed according to their relative abundance (false discovery rate <0.05). Inner to outer rings were organized following the order of phylum, class, order, family, and genus.

### Statistical Analysis

Statistical analysis was performed using SPSS 22.0. All experimental data are presented as means ± SEM. Comparisons within multiple groups were measured by ANOVA. *p* < 0.05 indicated statistical significance.

## Results

### QRXZF Decreased Urinary Albumin and Regulated Lipid Metabolism in DKD Mice

**Figure 1:**
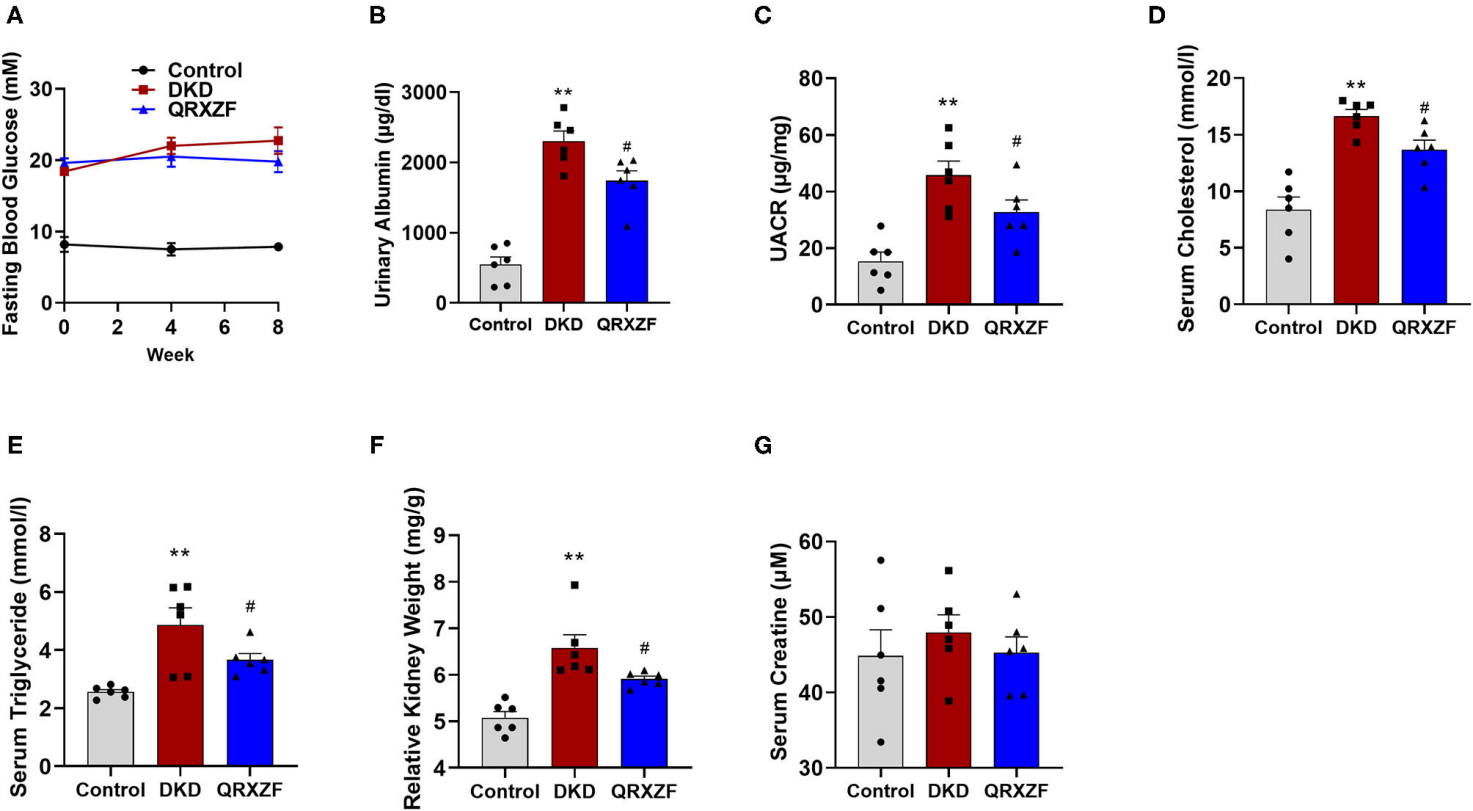
QRXZF decreased urinary albumin and ameliorated disorder of lipid metabolism in DKD mice after 8 weeks (*n* = 6, means ± SEM). **(A)** Serum glucose; **(B)** Urinary albumin content; **(C)** Urine albumin to creatinine ratio (UACR); **(D)** Serum cholesterol; **(E)** serum triglyceride; **(F)** Kidney weight/body weight; **(G)** Serum creatinine. ^*^p < 0.05, ^**^P < 0.01 DKD group vs. the control group; ^#^*p* < 0.05, ^##^*P* < 0.01 QRXZF group vs. the DKD group.

Diabetes was induced in HFD-fed mice after the STZ injection. These mice developed hyperglycemia at the first week (termed week 0) and high levels of blood glucose were maintained throughout the experiment. However, there were no significant differences in serum glucose between the QRXZF group and the DKD group at the end of both week 4 and week 8 (Figure 1A). Mice in the DKD group had remarkably increased urinary albumin content and urine albumin to creatinine ratio (UACR), which were reversed after treatment with QRXZF (Figures 1B,C). Similarly, mice in the DKD group had significantly higher levels of serum cholesterol and triglyceride, which were significantly reduced after treatment with QRXZF (Figures 1D,E). In addition, kidney weight/ body weight ratio of mice which received QRXZF were significantly lower than mice in the DKD group (Figure 1F). However, there were no significant differences of serum creatinine among the three groups, which may be because we only established an early stage DKD animal model (Figure 1G).

### QRXZF Attenuated Renal Injury and Inhibited Inflammatory Responses in the Kidney

**Figure 2:**
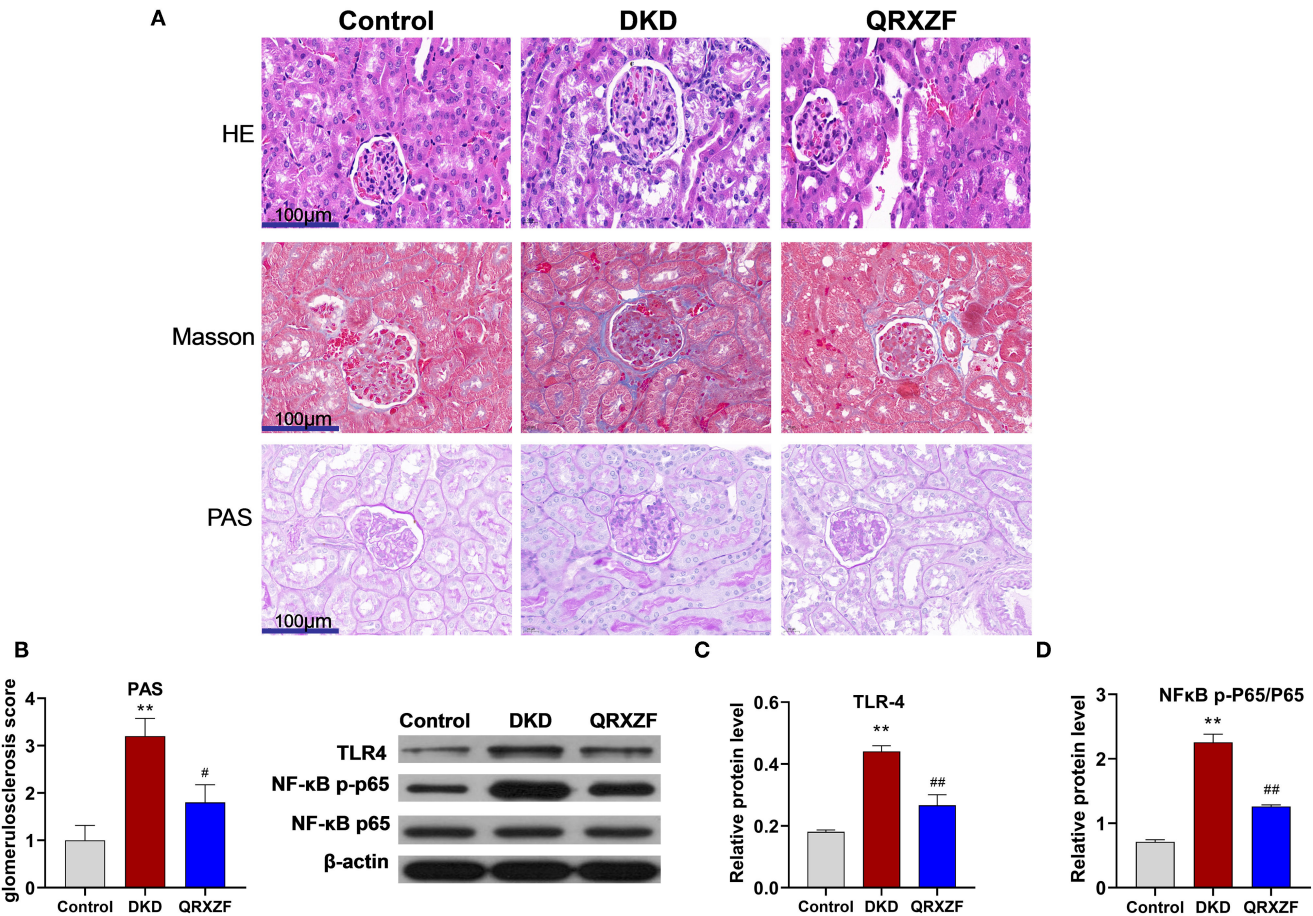
QRXZF attenuated renal injury and inhibited inflammatory response in the kidney. **(A)** HE, Masson, and PAS stained at 400×magnification, respectively; **(B)** PAS score; **(C,D)** western blot, expression of TLR4 and the ratio of NF-κB p-p65 to NF-κB p65 (*n* = 3, means ± SEM). ^*^p < 0.05, ^**^p < 0.01 DKD group vs. the control group; ^#^*p* < 0.05, ^##^*P* < 0.01 QRXZF group vs. the DKD group.

Histological features of kidneys from mice in the DKD group include glomerular hypertrophy, glomerular basement membrane (GBM) thickening, mesangial matrix expansion and vacuolar degeneration of tubular epithelial cells. After treatment with QRXZF, glomerular hypertrophy, mesangial matrix expansion and tubulointerstitial injury were partially ameliorated (Figure 2A). Sections stained using the Masson's trichrome stain showed that renal fibrosis was improved after QRXZF treatment (Figure 2A). In addition, and the PAS score to examine extracellular matrix (ECM) accumulation was also decreased after QRXZF treatment (Figures 2A,B).

The TLR4/NF-κB signaling pathway is crucial in the regulation of inflammation, while dysregulation might lead to higher levels of inflammation and subsequent DKD. As shown in the Western blot analysis, both the levels of TLR4 expression and the ratio of NF-κB p-p65 to NF-κB p65 were higher in the DKD group as compared to the control group (Figures 2C,D). In contrast, QRXZF inhibited the expression of TLR4 and reduced of the ratio of NF-κB p-p65 to NF-κBp65 in the kidney. These results show that QRXZF could suppressed the TLR4/NF-κB inflammation signaling pathway in the kidney.

### QRXZF Enhanced Intestinal Barrier Integrity

**Figure 3:**
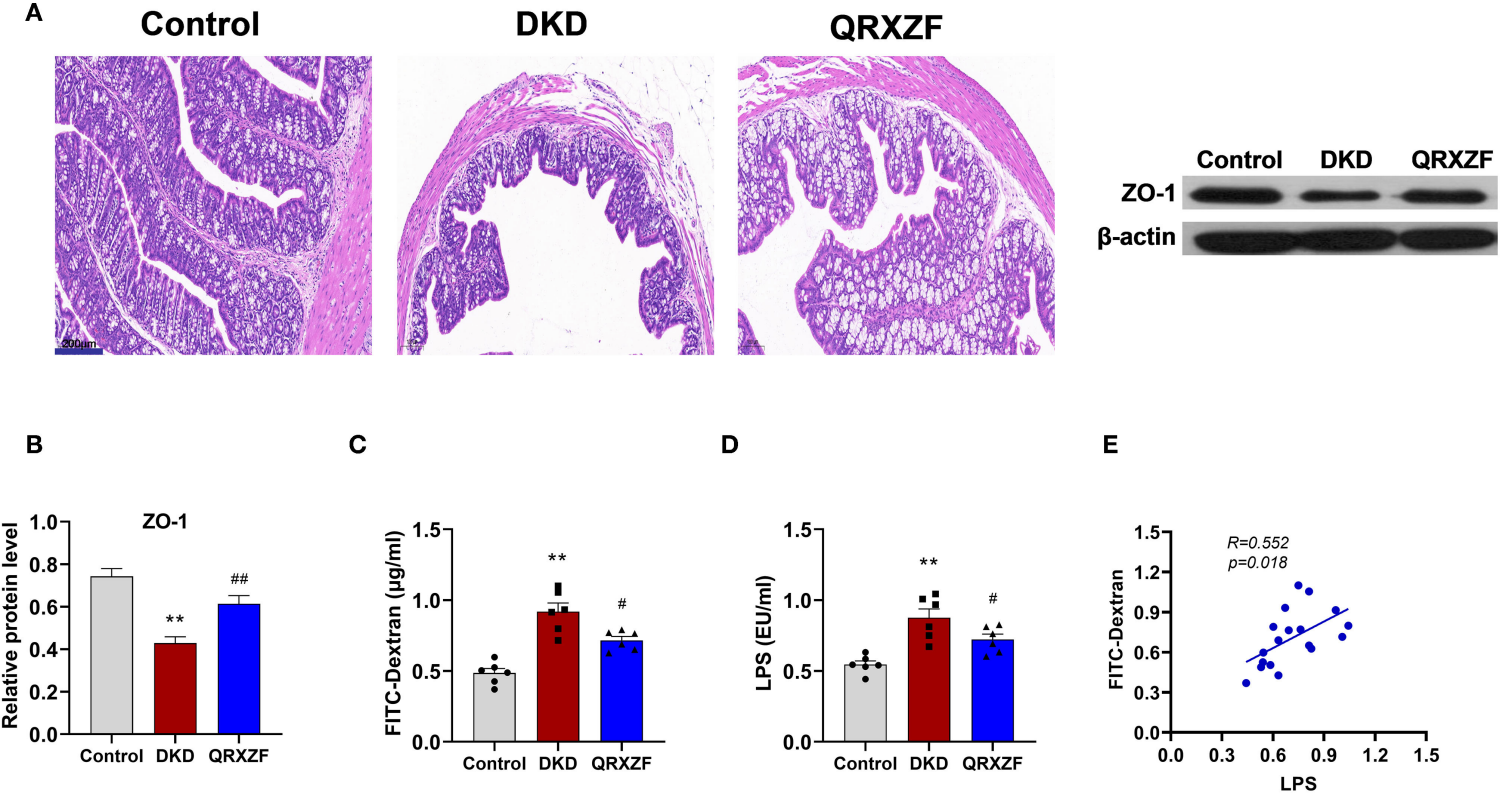
QRXZF improved gut barrier. **(A)** HE stained at 100×magnification; **(B)** Expression of ZO-1 evaluated by western blot analysis (*n* = 3, means ± SEM); **(C)** serum FITC-dextran (*n* = 6, means ± SEM.); **(D)** serum LPS; **(E)** Correlation analyses between serum LPS and FITC-dextran. ^*^p < 0.05, ^**^P < 0.01 DKD group vs. the control group; ^#^*p* < 0.05, ^##^*P* < 0.01 QRXZF group vs. the DKD group.

HE staining of colon tissue obtained from the DKD group showed greater damage to the intestinal mucosa as compared to the control group, which was ameliorated after QRXZF treatment (Figure 3A). Western blotting results showed that expression of ZO-1 protein in the colon was significantly upregulated after administration of QRXZF as compared with the DKD group (Figure 3B). Compared to the DKD group, levels of serum FITC-dextran, a marker of intestinal permeability was significantly lower in the QRXZF group (Figure 3C). Levels of serum LPS, an important indicator of inflammation, also decreased significantly after treatment with QRXZF (Figure 3D) and showed a positive correlation with levels of FITC-dextran (Figure 3E). These results show that QRXZF is effective in maintaining intestinal barrier integrity, which could be the reason for the reduced levels of circulating LPS.

### QRXZF Modulated the Gut Microbiota in DKD Mice

**Figure 4:**
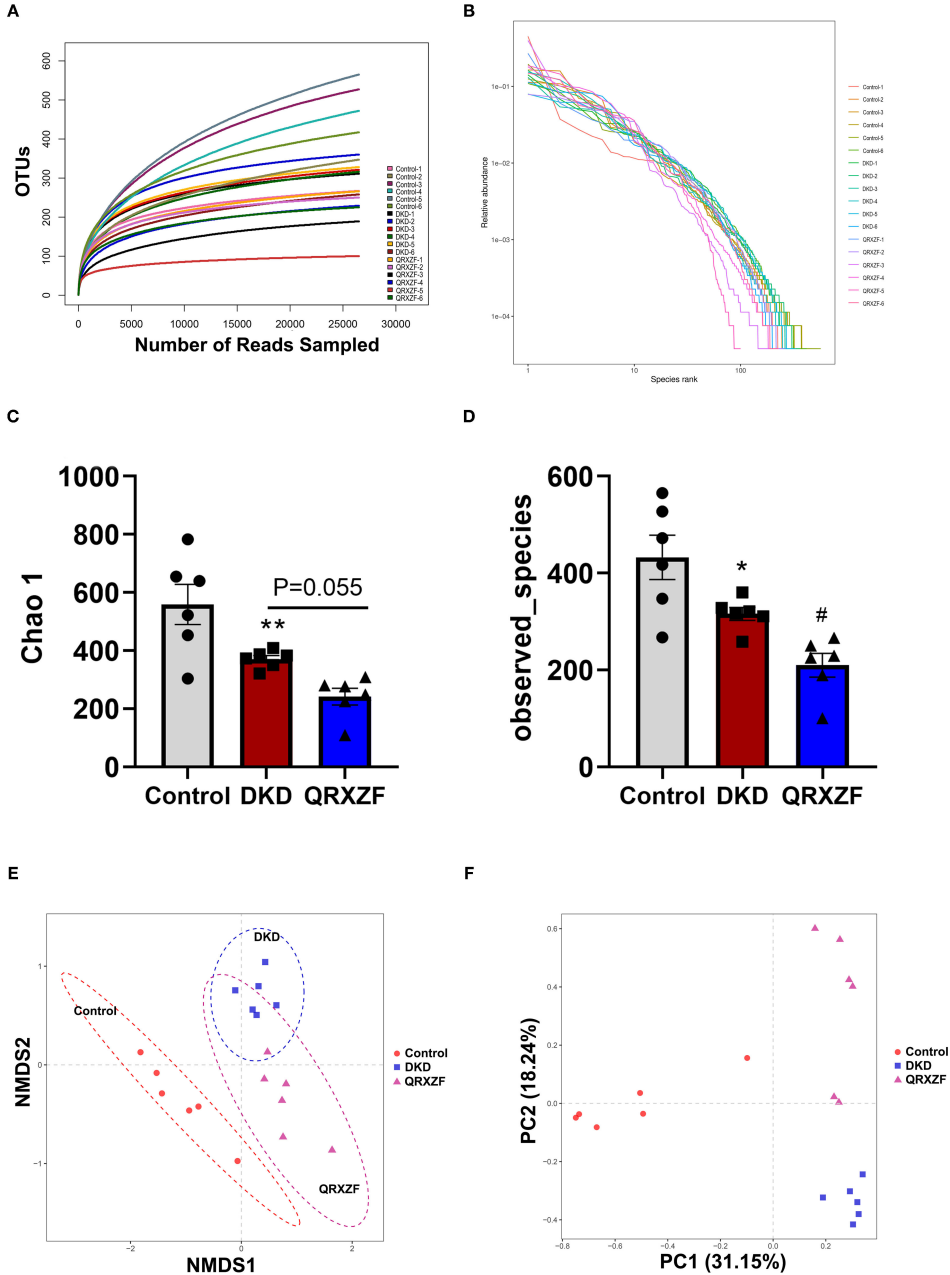
**(A)** Rarefaction curve; **(B)** Rank abundance curve; **(C,D)** Chao1 index and the observed species index (*n* = 6, means ± SEM); **(E)** Non-metric multidimensional scaling (NMDS) analysis between three groups; **(F)** Principal component analysis (PCA). ^*^p < 0.05, ^**^P < 0.01, ^***^P < 0.001 DKD group vs. the control group; ^#^*p* < 0.05, ^##^*P* < 0.01 QRXZF group vs. the DKD group.

When analyzing the composition of gut microbiota, sequences were divided into operational taxonomic units (OTUs) with a similarity cutoff of 97%. Rank abundance curves and rarefaction curves of each of the 18 samples being investigated plateaued with the depth of sequencing, indicating that the entire microbial community was captured (Figures 4A,B). The Chao 1 index and the observed species index selected to assess alpha diversity were significantly lower in the DKD group as compared to the control group. After treatment with QRXZF, both indices were further reduced in QRXZF group, though no significant differences were observed for the Chao 1 index (*P* = 0.055), suggesting that QRXZF treatment did not enrich the microbiota diversity (Figures 4C,D). Non-metric multidimensional scaling (NMDS) as well as principal component analysis (PCA) were conducted to assess beta diversity. Results indicated that the main components of the three groups could be well-distinguished and differences were identified in each group (Figures 4E,F).

**Figure 5:**
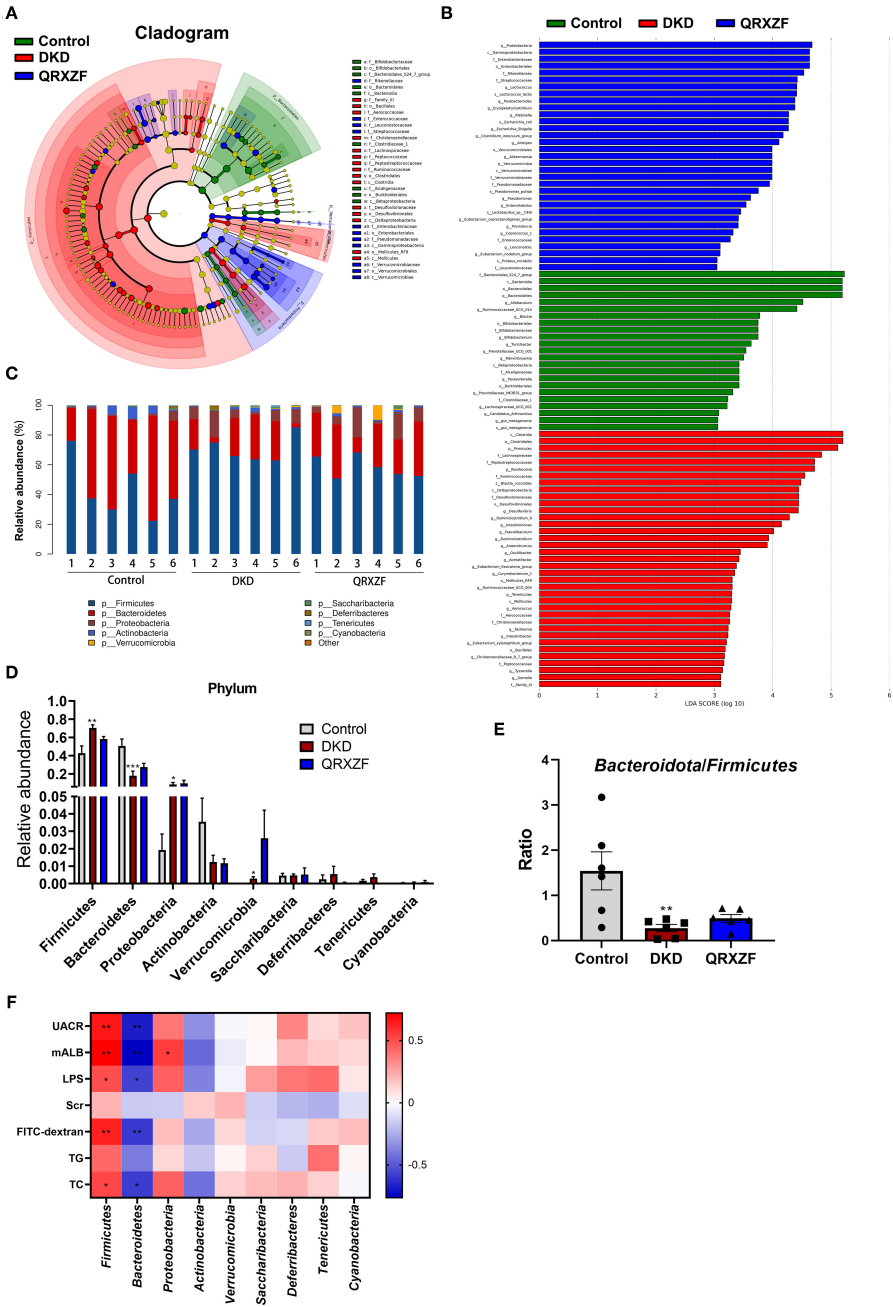
Key biomarkers of gut microbiota between each group. **(A)** Taxonomy analysis; **(B)** LEfSe analysis; **(C)** Relative abundance at phylum level; **(D)** Relative abundance at the phylum level between three groups; **(E)** *Bacteroidetes*-to-*Firmicutes* ratio (*n* = 6, means ± SEM); **(F)** Correlation analyses between metabolic parameters and relative abundance of gut microbiota at phylum level. ^*^p < 0.05, ^**^P < 0.01, ^***^P < 0.001 DKD group vs. the control group; ^#^*p* < 0.05, ^##^*P* < 0.01 QRXZF group vs. the DKD group; Colors ranged from blue to red, where blue indicates negative correlation and red indicates positive correlation. Significant correlations were marked by ^*^p < 0.05, ^**^P < 0.01.

To investigate the regulatory effect of QRXZF, a LEfSe analysis and cladogram were performed to reveal the dominant genera of the gut microbiota (Figures 5A,B). As shown in Figure 5C, the composition of gut microbiota of each group at the phylum level including *Bacteroidetes, Actinobacteria, Firmicutes, Proteobacteria, Saccharibacteria, Deferribacteres, Tenericutes*, and *Cyanobacteria. Firmicutes* and *Bacteroidetes* were the most dominant, with a total of >90%. As compared to the control group, the DKD group had a significantly lower abundance of *Bacteroidete* but higher abundance of *Firmicutes, Proteobacteria*, and *Verrucomicrobia* (Figure 5D). Ratio of *Bacteroidetes-to-Firmicutes* in the DKD group was also significantly decreased (Figure 5E).

We conducted Spearman's correlation analysis to establish correlation between parameters tested and relative abundances of gut microbiota at the phylum level. As shown in Figure 5F, results suggest that *Firmicutes* exhibited a positive correlation with UACR, microalbumin (mALB), TC, LPS and FITC-dextran, while *Bacteroidetes* had a negative correlation. *Proteobacteria* showed a positive correlation with mALB.

The abundance of *Desulfovibrionaceae* and *Desulfovibrio* were higher in the DKD group, which were closely related to increased leakage of LPS. Meanwhile, the DKD group had lower abundance of *Parasutterella*, a bacterium capable of producing short-chain fatty acids (SCFAs). SCFAs have anti-inflammatory and protective effects on the intestinal barrier. Mice treated with QRXZF had a higher abundance of *Rikenellaceae*, which might have enhanced the levels of SCFAs in the intestine.

Collectively, based on the spectrum of gut microbiota in the three groups, we speculated that injury to intestinal and renal tissues in the DKD group was related to the increased LPS-releasing bacteria but decreased the levels of bacteria with gut protective effects. QRXZF could protect the intestinal barrier, reduce LPS and kidney injury which may be related to changes of gut microbiota.

**Figure 6:**
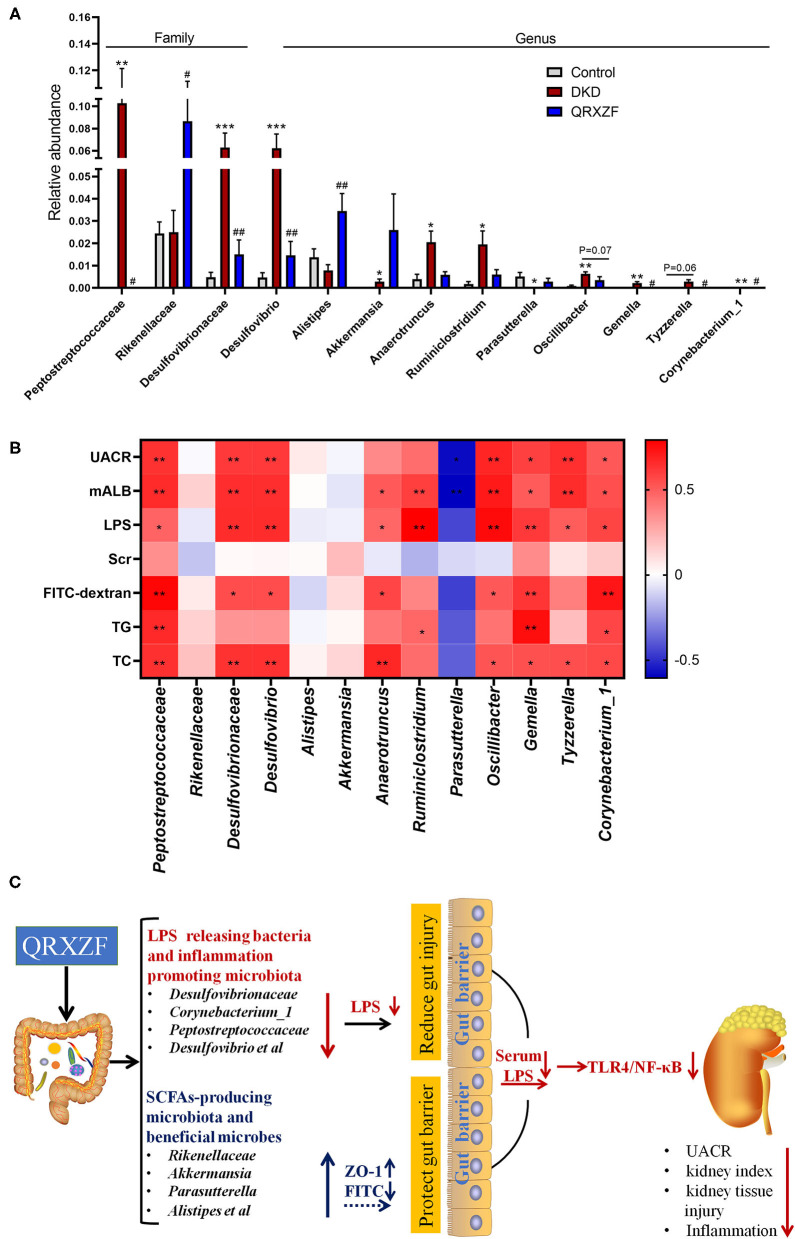
**(A)** Relative abundance at the family and genus level (*n* = 6, means ± SEM); **(B)** Correlation analyses between metabolic parameters and relative abundance of gut microbiota at family and genus level; **(C)** The mechanisms schematic of action of QRXZF in High-fat diet (HFD) and streptozotocin injection-induced DKD mice model. QRXZF treatment could prevent the gut dysbiosis, reduce the intestinal permeability and gut-derived LPS into blood. The reno-protective effect of QRXZF might be associated with inhibition of the LPS/TLR4/NF-κB inflammation signaling pathway in the kidney. ^*^p < 0.05, ^**^P < 0.01, ^***^P < 0.001 DKD group vs. the control group; ^#^*p* < 0.05, ^##^*P* < 0.01 QRXZF group vs. the DKD group; Colors ranged from blue to red, where blue indicates negative correlation and red indicates positive correlation. Significant correlations were marked by ^*^p < 0.05, ^**^P < 0.01.

The results showed that *Peptostreptococcaceae, Rikenellaceae, Desulfovibrionaceae, Desulfovibrio, Corynebacterium_1, Anaerotruncus, Gemella, Tyzzerella, Oscillibacter, Ruminiclostridium, Parasutterella, Alistipes* and *Akkermansia* at the family and genus levels were selected to be meaningful gut microbiota (Figure 6A). Spearman's correlation analysis suggested that *Peptostreptococcaceae, Gemella* and *Corynebacterium_1* exhibited a positive correlation with levels of UACR, mALB, LPS, FITC-dextran, TG and TC. *Desulfovibrionaceae, Desulfovibrio* and *Oscillibacter* exhibited a positive correlation with levels of UACR, mALB, LPS, FITC-dextran and TC. *Anaerotruncus* exhibited a positive correlation with levels of mALB, LPS, FITC-dextran and TC. *Ruminiclostridium* exhibited a positive correlation with levels of mALB, LPS and TG. *Tyzzerella* exhibited a positive correlation with levels of UACR, mALB, LPS and TC. However, *Parasutterella* have negative correlations with levels of UACR and mALB, respectively (Figure 6B).

## Discussion

DKD is the main cause of ESRD. As DKD progresses, it results in glomerular hyperfiltration, increasing albuminuria, and declining estimated glomerular filtration rate (eGFR), which ultimately leads to ESRD (21). In this study, we successfully induced an appropriate mouse model with early-stage DKD through HFD feeding followed by low-dose STZ injection. The DKD mice exhibited a rise in blood glucose levels, weight/body weight ratio, UACR and more severe pathological damage of kidney tissue. Excepting of blood glucose, these indices were improved after treatment with QRXZF. In addition, QRXZF was also effective in improving lipid metabolism.

Evidence show that dysbiosis of gut microbiota could lead to metabolic diseases and kidney disease (22, 23). Our results showed that there were significant differences in alpha and beta diversity for three groups. These results indicate that QRXZF had significant effects on the diversity of gut microbiota. *Firmicutes* and *Bacteroidetes* were the dominant gut microbiota at phyla level (24). In our study, the DKD group had a significantly higher relative abundance of *Firmicutes*, but lower abundance of *Bacteroidetes* as well as the ratio of *Bacteroidetes* to *Firmicutes*. These changes were closely related to HFD (25), as well as obesity and lipid deposition (26). Composition of gut microbiota was altered after treatment with QRXZF, which could have resulted in improved regulation of lipid metabolism.

At the family and genus level, differences in gut microbiota exist amongst three groups. The abundance of *Desulfovibrionaceae, Desulfovibrio, Peptostreptococcaceae, Corynebacterium_1* was higher in the DKD group. Desulfovbrionaceae and *Desulfovibrio* are LPS-producing bacteria (27, 28), where LPS produced by Desulfovbrionaceae have potent inflammation-inducing capacities, usually 100- to 1,000-fold higher than LPS from *Bacteroides spp* (29), which are involved in gut permeability and chronic inflammation (30). As a potential human pathogen, *Corynebacterium_1* could enhance an individual's susceptibility of LPS (31) and increase of the levels of inflammation (32). *Peptostreptococcaceae*, a bacterium that promotes inflammation, was more abundant in the DKD group (33).

Additionally, *Anaerotruncus, Gemella, Tyzzerella, Oscillibacter*, and *Ruminiclostridium* were more abundant in the DKD group as compared to the control group, while *Parasutterella* and *Alistipes* were less abundant. *Alistipes* and *Parasutterella* (23, 34) synthesize SCFAs. while *Anaerotruncus, Tyzzerella* and *Gemella* were negatively correlated with levels of plasma SCFAs (35). SCFAs are an essential source of energy and contribute to gut barrier integrity (36, 37), down-regulation of inflammatory factors and inhibition of kidney inflammation (38, 39). *Oscillibacter* and *Ruminiclostridium* were negatively correlated with the expression of ZO-1 protein, a protein important for the maintenance of gut barrier (40–42). However, the reason for this is unclear. We postulate that *Oscillibacter* and *Ruminiclostridium* could possibly regulate mechanisms associated with gut barrier integrity or it could alter the composition of gut microbiota, leading to changes in the levels of ZO-1 protein. After treatment with QRXZF, almost all of the above results were reversed.

After administration of QRXZF, the abundance of *Rikenellaceae* and *Akkermansia* were enriched. *Rikenellaceae* are also positively correlated with the production of butyric and valeric acids, which are important component of SCFAs (43). *Akkermansia* are beneficial microbes (44), which could reduce levels of serum LPS, relieve intestinal mucosal damage and contribute to better metabolism (45, 46). Taken together, our findings suggest that QRXZF could decrease LPS-producing microbiota and increase SCFAs-producing microbiota as well as gut barrier protective microbiota, which suggests that QRXZF has positive effects on gut dysbiosis and the gut barrier function.

DKD is a chronic inflammatory disease accompanied by lipid disorders (47, 48). Evidence shows that gut-derived LPS may be crucial in chronic inflammation and progression of DKD (14, 17). In our study, we have shown that damage to the gut barrier led to a lower expression of tight junction proteins ZO-1, higher levels of the serum FITC-dextran and subsequently increased intestinal permeability, which may facilitate the passage of gut-derived LPS into the blood. Gut-derived LPS could initiate inflammatory responses through TLRs, in particular through the TLR4-related pathway, where LPS mediates the activation of NF-κB (13). This would cause chronic inflammation and accelerate DKD. Evidence also shows that the TLR4/NF-κB pathways in the kidney are closely related to the development of DKD (49, 50). Our study shows that the LPS/TLR4/NF-κB pathway was up-regulated in DKD group, as we found increased levels of serum LPS and overexpression of TLR4 and NF-κB in the kidney. However, the LPS/TLR4/NF-κB pathway was downregulated after QRXZF treatment.

Besides, to further test our hypothesis that the gut microbiota and the inflammatory responses were inevitable correlation with the reno-protective effect of QRXZF against DKD, the fecal microbiota transplantation (FMT) (51, 52) experiment or germ-free mice would be performed in further studies to provide more evidences about QRXZF.

## Conclusion

Our study demonstrates that QRXZF could prevent the gut dysbiosis, reduce the intestinal permeability and gut-derived LPS into blood. The reno-protective effect of QRXZF might be associated with inhibition of the LPS/TLR4/NF-κB inflammation signaling pathway in the kidney (Figure 6C).

## Data Availability Statement

The datasets presented in this study can be found in online repositories. The names of the repository/repositories and accession number(s) can be found below: https://www.ncbi.nlm.nih.gov/, PRJNA729207.

## Ethics Statement

The animal study was reviewed and approved by Ethics Committee of Beijing University of TCM.

## Author Contributions

ZW and LP: conceptualization, funding acquisition, and writing—review and editing. YW and WL: supervision. YG, RY, ZW, and YZ: formal analysis, investigation, writing—original draft, and visualization. SA and TW: writing—review editing. All authors approved of the final submission.

## Funding

This study was supported by the National Natural Science Foundation of China (81804032, 81904105, and 81970713), Research Start up Fund for Doctor of Medicine of the First Affiliated Hospital of Henan University of Traditional Chinese Medicine (2021BSJJ021), and the top talent training program of Henan Province of traditional Chinese Medicine.

## Conflict of Interest

The authors declare that the research was conducted in the absence of any commercial or financial relationships that could be construed as a potential conflict of interest.

## Publisher's Note

All claims expressed in this article are solely those of the authors and do not necessarily represent those of their affiliated organizations, or those of the publisher, the editors and the reviewers. Any product that may be evaluated in this article, or claim that may be made by its manufacturer, is not guaranteed or endorsed by the publisher.
