# Supplementary figures and images for: Qing-Re-Xiao-Zheng Formula Modulates Gut Microbiota and Inhibits Inflammation in Mice With Diabetic Kidney Disease
Source: Front Med (Lausanne). 2021 Sep 16;8:719950. doi: 10.3389/fmed.2021.719950 (PMC8481597; doi:10.3389/fmed.2021.719950)

## Slide 1
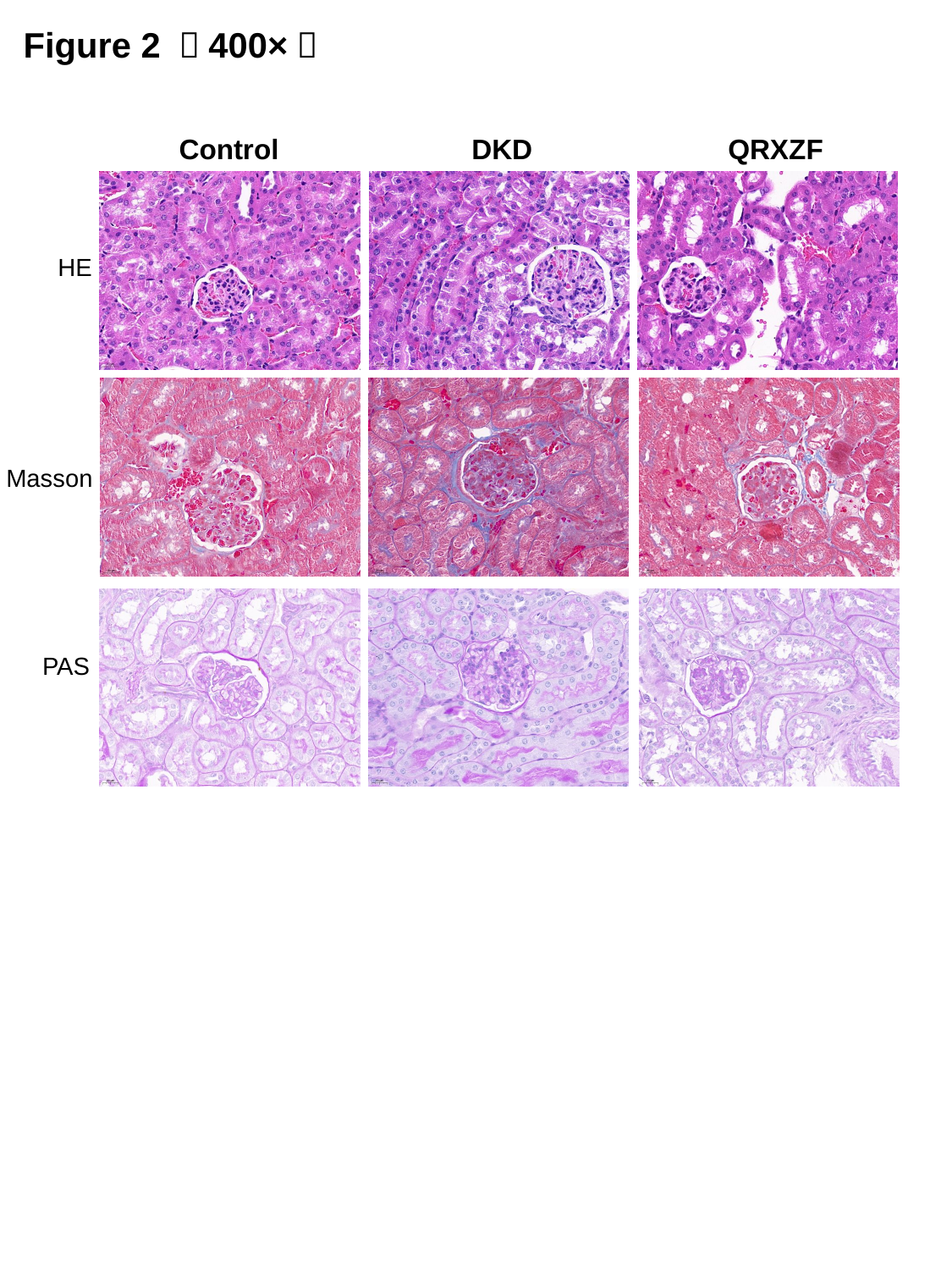

Figure 2 （400×）
Control
DKD
QRXZF
HE
Masson
PAS

## Slide 2
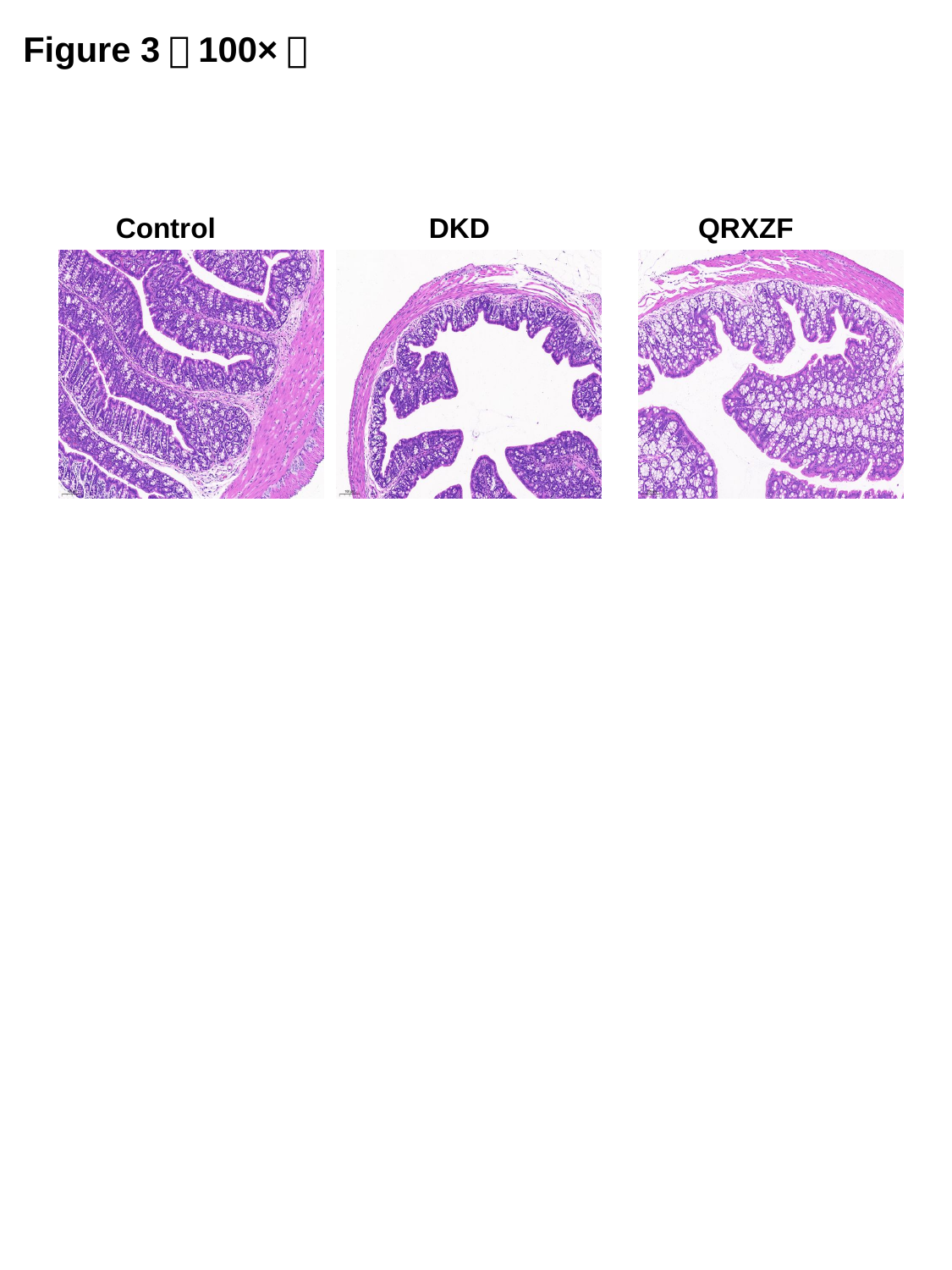

Figure 3（100×）
Control
DKD
QRXZF

Supplement: Supplementary file 7 [file Presentation_1.PPT]
