# Supplementary material for: Qing-Re-Xiao-Zheng Formula Modulates Gut Microbiota and Inhibits Inflammation in Mice With Diabetic Kidney Disease
Source: Front Med (Lausanne). 2021 Sep 16;8:719950. doi: 10.3389/fmed.2021.719950 (PMC8481597; doi:10.3389/fmed.2021.719950)
Supplement: Supplementary file 8 [file Presentation_2.PPT]

## Slide 1
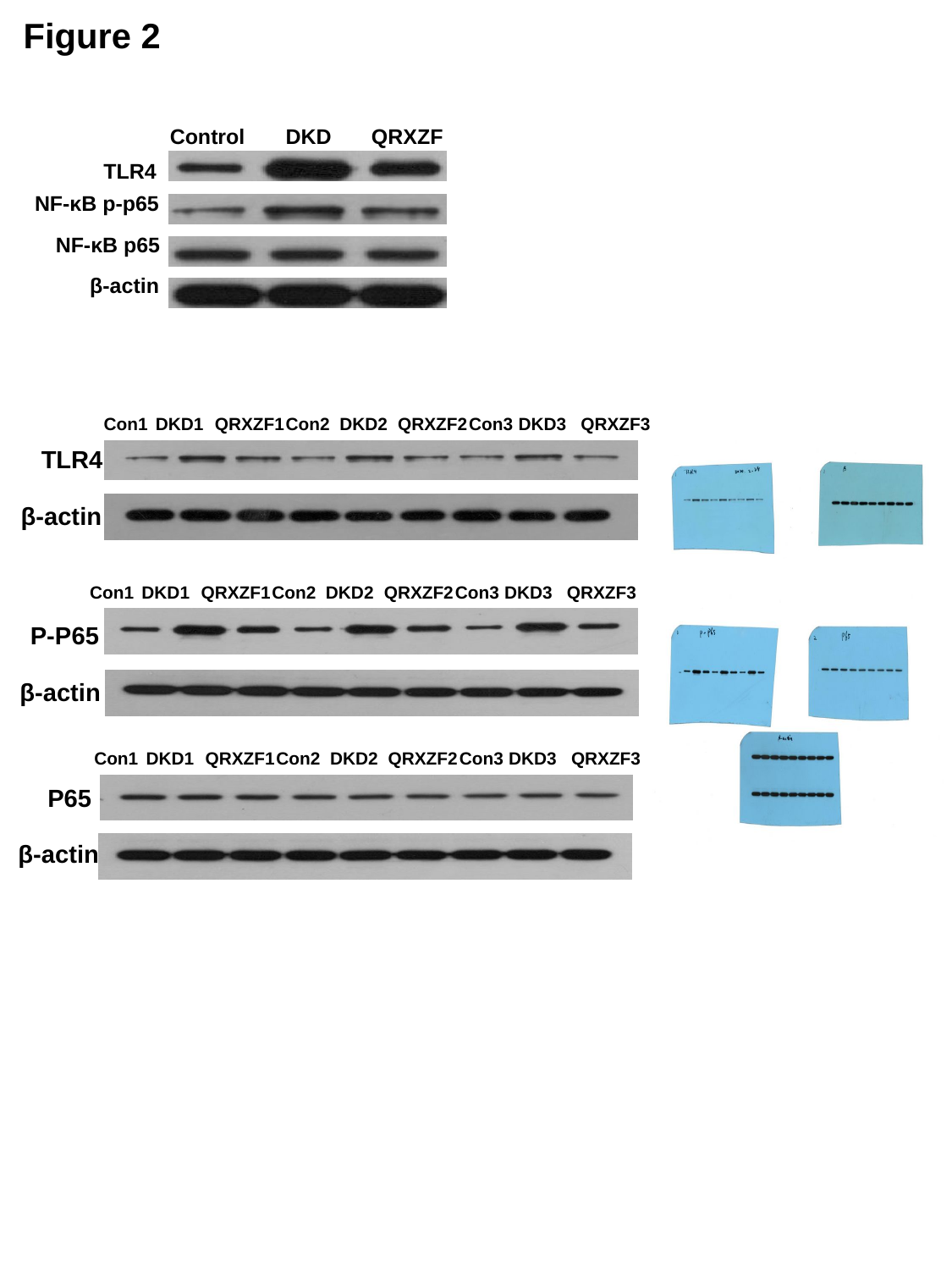

Figure 2
Control
DKD
QRXZF
TLR4
NF-κB p-p65
NF-κB p65
β-actin
Con1
DKD1
QRXZF1
Con2
DKD2
QRXZF2
Con3
DKD3
QRXZF3
TLR4
β-actin
P-P65
β-actin
P65
β-actin
Con1
DKD1
QRXZF1
Con2
DKD2
QRXZF2
Con3
DKD3
QRXZF3
Con1
DKD1
QRXZF1
Con2
DKD2
QRXZF2
Con3
DKD3
QRXZF3

## Slide 2
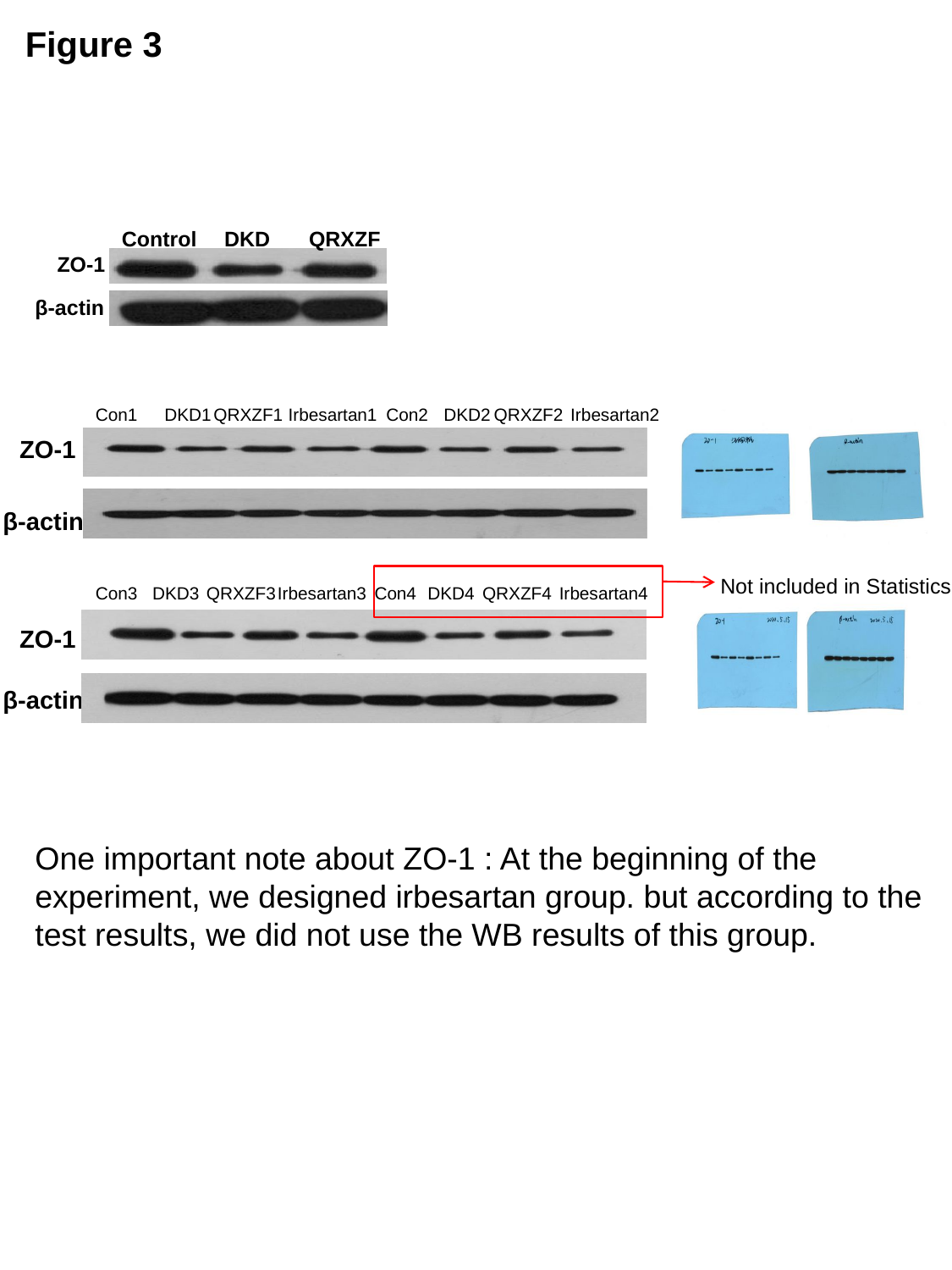

Figure 3
Control
DKD
QRXZF
ZO-1
β-actin
Con1
DKD1
QRXZF1
Irbesartan1
Con2
DKD2
QRXZF2
Irbesartan2
ZO-1
β-actin
Con3
DKD3
QRXZF3
Irbesartan3
Con4
DKD4
QRXZF4
Irbesartan4
ZO-1
β-actin
Not included in Statistics
One important note about ZO-1 : At the beginning of the experiment, we designed irbesartan group. but according to the test results, we did not use the WB results of this group.
