# Supplementary material for: Qing-Re-Xiao-Zheng Formula Modulates Gut Microbiota and Inhibits Inflammation in Mice With Diabetic Kidney Disease
Source: Front Med (Lausanne). 2021 Sep 16;8:719950. doi: 10.3389/fmed.2021.719950 (PMC8481597; doi:10.3389/fmed.2021.719950)
Supplement: Supplementary file 9 [file Presentation_3.PPT]

## Slide 1
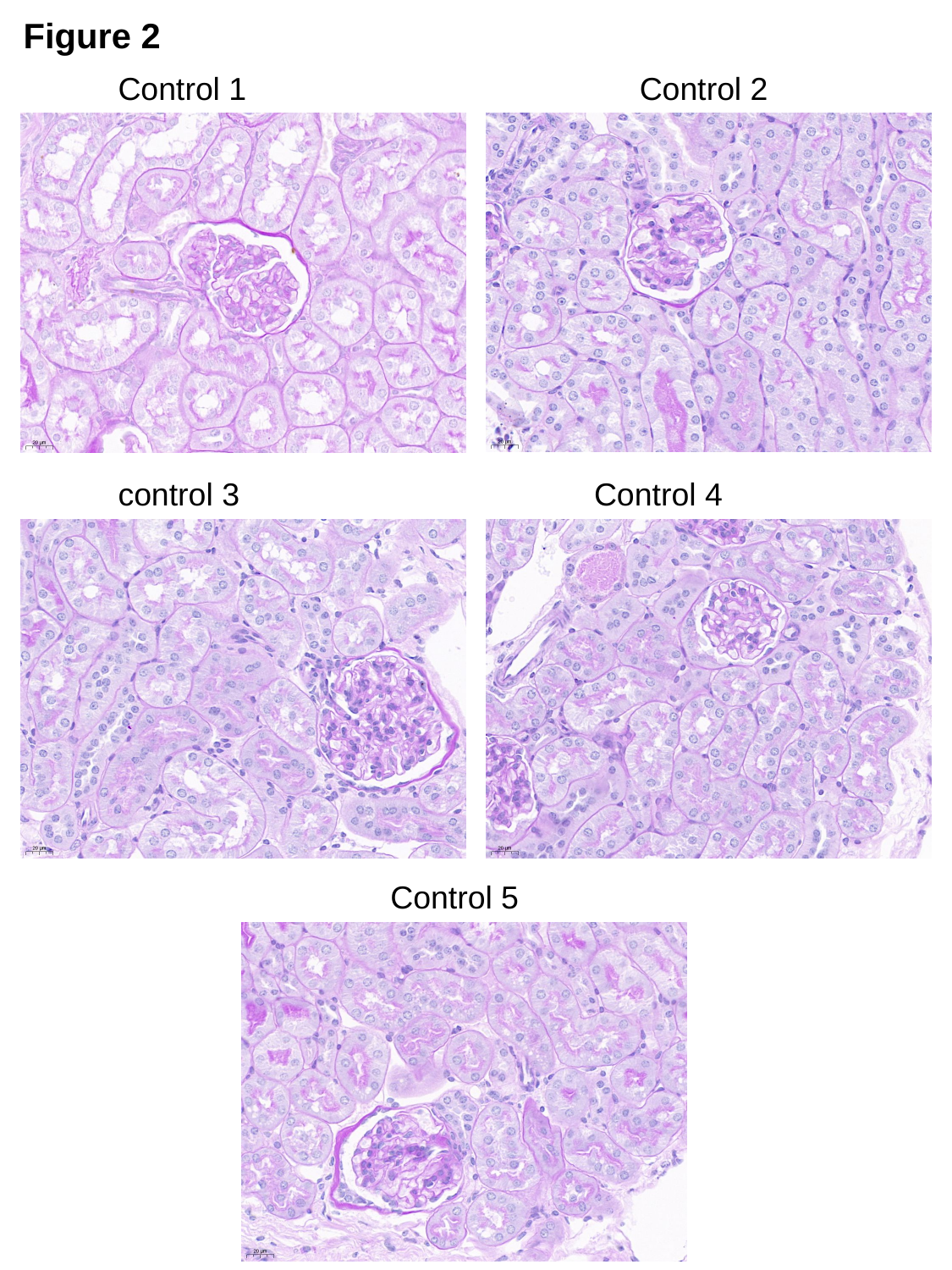

Figure 2
Control 1
Control 2
control 3
Control 4
Control 5

## Slide 2
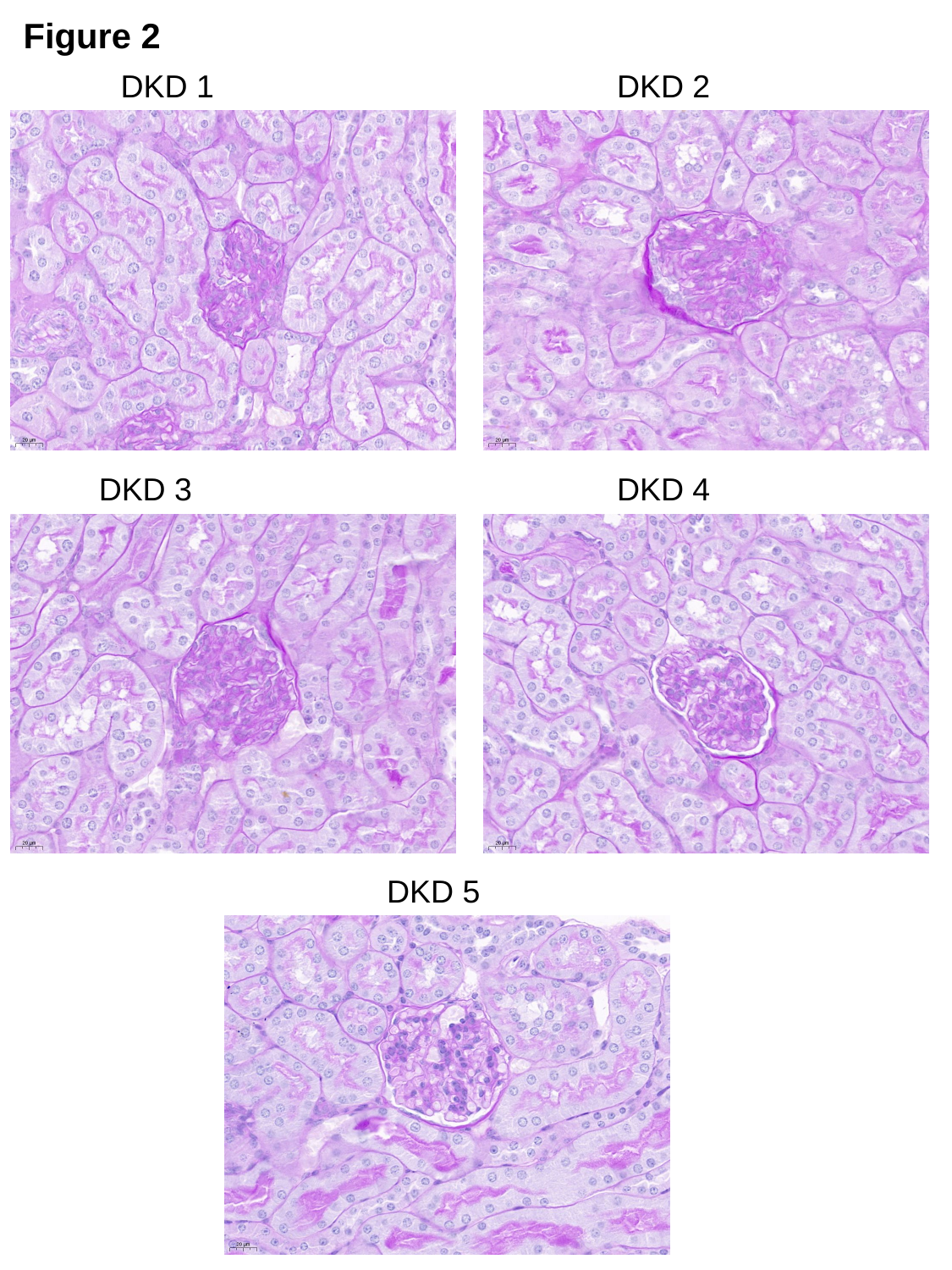

Figure 2
DKD 1
DKD 2
DKD 3
DKD 4
DKD 5

## Slide 3
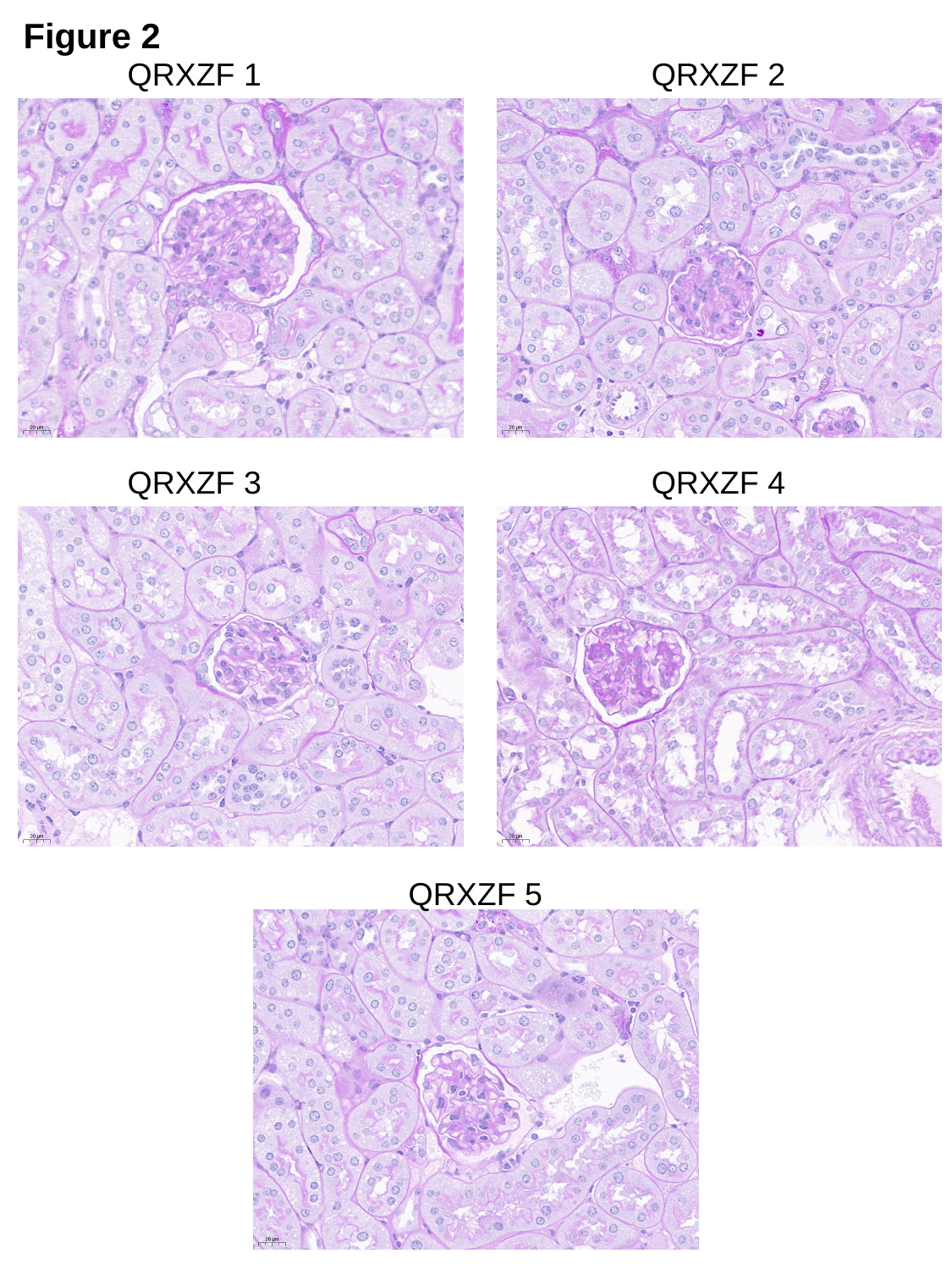

Figure 2
QRXZF 1
QRXZF 2
QRXZF 3
QRXZF 4
QRXZF 5
